# Epidemiological and Clinical Characteristics of 5,569 Pediatric Burns in Central China From 2013 to 2019

**DOI:** 10.3389/fpubh.2022.751615

**Published:** 2022-03-29

**Authors:** Dawei Han, Ying Wei, Yancang Li, Xinjian Zha, Rui Li, Chengde Xia, Yun Li, Huanna Yang, Jiangfan Xie, Shemin Tian

**Affiliations:** ^1^Department of Burns, Zhengzhou First People's Hospital, Zhengzhou, China; ^2^Department of Radiology, The First Affiliated Hospital of Zhengzhou University, Zhengzhou, China

**Keywords:** epidemiology, pediatric, burns, outcome, prevention, cost

## Abstract

**Background:**

Pediatric burns of all the ages are prevalent worldwide, posing a severe health risk to children. This study aims to examine pediatric burns' clinical characteristics and epidemiology in central China.

**Methods:**

The pediatric patients of the Burn Research Center, Department of the First People's Hospital of Zhengzhou City from 2013 to 2019 were retrospectively studied and the relevant data were collected from the hospitalized medical records [e.g., demographic, etiology, length of stay (LOS), age, gender, burn area and depth, number of surgeries, cost, and outcome].

**Results:**

A total of 5,569 pediatric burn patients were included, accounting for 43.9% of the total burn population. Electric burns represented a relatively small proportion (1.17%) but were more likely to lead to disabilities or death than scalds (90.63%) and flames (5.12%). The median age was 2 years [interquartile range (IQR): 1–4] and the boys/girls ratio ranged from 1.3:1 to 1.6:1. The most commonly burnt anatomic sites were the limbs (38.3%), with a median %TBSA (total body surface area) of 6 (IQR: 4–10). The complications of shock and pneumonia accounted for 7.6 and 19.2%, respectively. The peak months of pediatric burns included January, May, and August and the rural/urban ratio reached 1.61:1. The percentage of burn wounds treated surgically increased considerably from 2013 to 2019 (3.8 vs. 37.8%). The median hospital LOS was 15 days (IQR: 8–28 days), with the three high-risk factors (e.g., more surgeries, more %TBSA, full-thickness skin burns). The median cost of hospitalization was 1,511 USD (IQR: 848–2,648 USD) and the main risk factors consisted of full-thickness burns, more %TBSA, longer LOS, and more surgical procedures. Among all the patients, LA50 was 78.63% (95% CI = 75.12–83.45) and the overall mortality reached 0.1% since seven deaths were recorded.

**Conclusion:**

Scalds, flames, contact, and chemicals are the main causes of burns among children aged 1–5 years in central China. Accordingly, various prevention strategies should be employed depending upon the cause of the burn.

## Introduction

Burns have become the fifth most common cause of nonfatal injuries to children (1). Globally, burns have gradually become a hotspot of public health concern, not just affecting any particular population or region (1–5). Moreover, in hospitalized burn patients, those aged 0 to 5 years represent the highest percentage (6). Scald is the most common type of pediatric burn, followed by flame, contact, chemical, etc. (4). The incidence of burns seems to be closely related to a region's economic development. Pediatric burn occurs much higher in low- or middle-income nations (e.g., Southeast Asia and Africa), whereas it has been declining in the United States over the past decade (7), which is consistent with the previous reports from north China (8), southwest China (9), and south-central China (10). Nevertheless, no relevant reports remain on central China, which prompted us to conduct this survey.

The Burn Research Institute of Zhengzhou First People's Hospital is regarded as an essential center in central China for burn diagnosis and treatment, which comprises 136 beds [includes 10 intensive care unit (ICU) beds]. The institute is located in Henan Province. The large population of over 100 million in this province results in a relatively high incidence of burns. This study primarily involves patients from Henan Province, western Anhui Province, southwestern Shanxi Province, etc. Though pediatric burns account for more than 43% each year, epidemiological investigations and studies have been rare. Using specific epidemiology as the basis, we can effectively prevent pediatric burns, increase the cure rate, and reduce medical expenses (11). To further improve the effectiveness of prevention strategies and therapeutic schemes in central China, the epidemiology and treatment of burns in children hospitalized between January 2013 and December 2019 should be carefully examined.

## Methods

From 2013 to 2019, data were collected on 5,569 burn children hospitalized at the Burn Research Center of Zhengzhou First People's Hospital. In addition, all the data were extracted from the electronic medical record system. The data consisted of ages, genders, dates of injury, dates of admission, burn etiology, %TBSA, burn depth and site, inhalation injuries, number of surgeries, and medical costs. Moreover, clinical outcome, length of stay (LOS), and date of hospital discharge were noted. Based on the collected data, the Burn Index (BI) (12) and the modified Baux score (13) were calculated. The Abbreviated Burn Severity Index (ABSI) (14) scoring system was cited for all the cases to achieve more rigorous results. The results of patient treatment are presented below. As an outcome, “healing” was defined as the wound completely healing with no remnant burned area remaining; as “improvement” for reduced, but not cleared burn areas; as “ineffectiveness” for exacerbation of the burn; and as “death” for the patient's condition worsening and dying (9). Pediatric burns are at high risk of shock, if the following factors are present: a burned area of 10% TBSA or greater, the third-degree burn area of 2% or greater, inhalation injury or exceedingly deep burns (e.g., 4th-degree injuries), and urine output < 1 ml/kg/h (15–17). Pneumonia is most commonly diagnosed in patients who have fever, cough, tachypnea, rales, chest radiographic consolidation, or infiltrates (18).

The Ethical Review Committee of Zhengzhou First People's Hospital has approved this article concerning a longitudinal and retrospective study. Informed consent was not required on account of the study's retrospective nature.

### Statistical Analysis

Microsoft Excel 2010 (Microsoft) was used to organize and categorize data, while calculating the descriptive data [e.g., median, SD, average, interquartile range (IQR)]. In addition, GraphPad Prism 8 (GraphPad Software Incorporation, USA) and SPSS version 20.0 software (IBM Corporation, USA) were used to analyze the statistics. The chi-squared test, the Mann–Whitney *U*-test, and the Kruskal–Wallis test were performed to detect the different groups of nonnormally distributed categorical or quantitative variables. Furthermore, the Dunn's test was performed *post-hoc* to compare the two groups. Using the Fisher's exact test for the chi-squared test if the theoretical frequency is <5 or total samples are fewer than 40. ANOVA with one way and Scheffé's test were conducted to compare mean differences between groups of quantitative variables with normal distributions. Multiple linear regression was utilized to analyze the risk factors associated with LOS and total medical costs. Statistical significance is determined by a *P* < 0.05.

## Results

### General Characteristics

**Figure 1:**
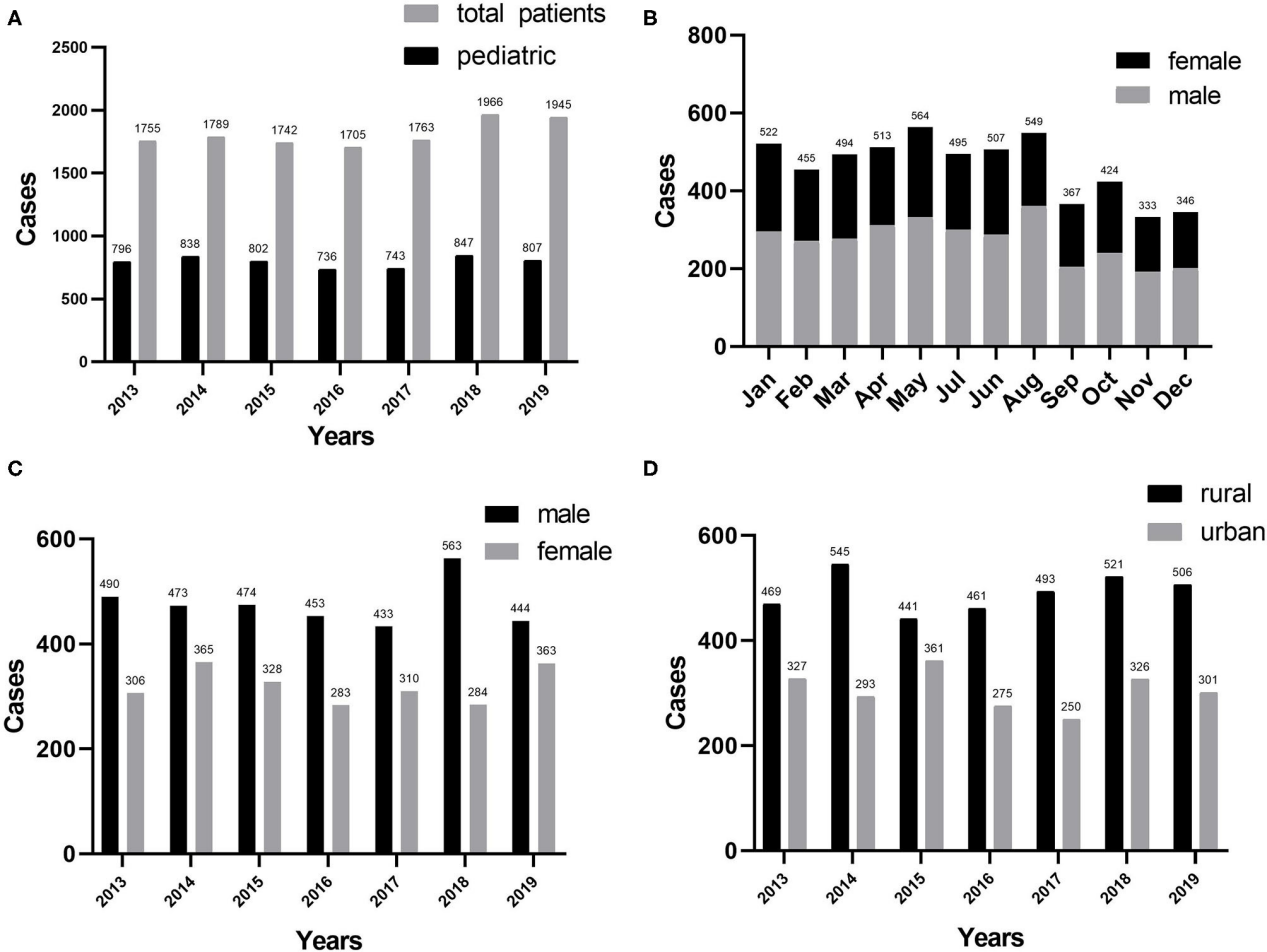
**(A)** Distribution of pediatric burns (*n* = 5,569) by years. **(B)** Pediatric burns by month. **(C)** Distribution of gender by years. **(D)** Regional distribution by years.

Table 1 summarizes the general characteristics of all the inpatients enrolled in this study. In total, 5,569 pediatric patients in the burn center were investigated for this study from January 2013 to December 2019 (43.9% of 12,665 patients). In 7 years, no significant difference has been observed in the number of burn patients hospitalized. In general, the average age of all the pediatric burn patients was 3.72 ± 3.05 years, ranging from 1 month to 14 years. Incidence of burns peaked in 2018 and nadir in 2016 (Figure 1A). Burns peaked in January, May, and August, whereas they declined to a minimum in November and December (Figure 1B). Over the past 7 years, the gender ratio has been relatively constant at 1.4:1. The ratio reached its maximum in 2016 (Figure 1C). Approximately, 7.6% of all the inpatients were accompanied by shock, with the maximum incidence occurring in 2015. Furthermore, pneumonia was the most common postinjury morbidity, with the highest percentage found in 2017. The median LOS was 15 days, ranging from 1 to 90 days. Overall, the cure and response rates were 86.3 and 98.5%, respectively. Surgery rates for all the inpatients were 13.3% and the number of pediatric burn surgeries increased significantly from 2013 to 2019. Seven deaths were reported from all the patients, accounting for 0.1% of the total mortality.

**Table 1:** Patient characteristics from 2013 to 2019.

**Years**	**Pediatric^**a**^**	**Age** **median (IQR)**	**Boys /Girls**	**Shock** ***N*** **(%)**	**Pneumonia *N* (%)**	**LOS** **median (IQR)**	**Cure rate** ***N*** **(%)**	**Response rate** ***N*** **(%)**	**Surgery** ***N*** **(%)**	**Mortality** ***N*** **(%)**
2013	796 (44.7)	2 (1–3)	1.3:1	51 (6.4)	153 (19.2)	12 (7–12)	693 (87.1)	784 (98.5)	30 (3.8)	0 (0)
2014	838 (46.8)	2 (1–4)	1.4:1	53 (6.3)	135 (16.1)	13 (8–22)	681 (81.3)	827 (98.7)	45 (5.4)	1 (0.1)
2015	802 (46.0)	2 (1–4)	1.3:1	74 (9.2)	155 (19.3)	13 (8–22)	731 (91.1)	793 (98.9)	106 (13.2)	1 (0.1)
2016	736 (43.6)	2 (1–3)	1.6:1	59 (8.0)	121 (16.4)	14 (9–25)	614 (83.4)	722 (98.1)	89 (12.0)	0 (0)
2017	743 (42.1)	2 (1–3)	1.4:1	55 (7.4)	178 (24.0)	18 (10–31)	647 (87.1)	736 (99.1)	112 (15.0)	2 (0.3)
2018	847 (43.1)	2 (1–4)	1.5:1	69 (7.4)	143 (16.9)	17 (10–31)	736 (86.8)	835 (98.6)	121 (14.3)	2 (0.2)
2019	807 (41.5)	2 (1–4)	1.4:1	62 (7.7)	185 (22.9)	16 (10–28)	706 (87.5)	788 (97.6)	305 (37.8)	1 (0.1)
total	5,569 (43.9)	2 (1–4)	1.4:1	423 (7.6)	1,070 (19.2)	15 (8–28)	4,808 (86.3)	5,485 (98.5)	808 (13.3)	7 (0.1)
P-value	0.005	0.314	1.00	0.305	<0.001	0.02	<0.001	0.291	<0.001	0.767

a *Hospitalized pediatric burns of 0–14 years old (exclusion of patients discharged within 24 h). IQR, Interquartile range*.

### Residential Areas

The prevalence of pediatric burns in different habitation areas in the same geographical area varied markedly, with 38.30% in the urban area and 61.70% in the rural area (using the residential address to distinguish rural from urban). Approximately, 1.97:1 was the maximum rural–urban ratio for 2017, 1.22:1 was the minimum rural–urban ratio for 2015, and the average ratio over the past 7 years was 1.61:1. The incidence of burns among children in the rural and urban areas differed significantly (*P* < 0.05) (Figure 1D).

### Etiology of Burn Injuries

**Figure 2:**
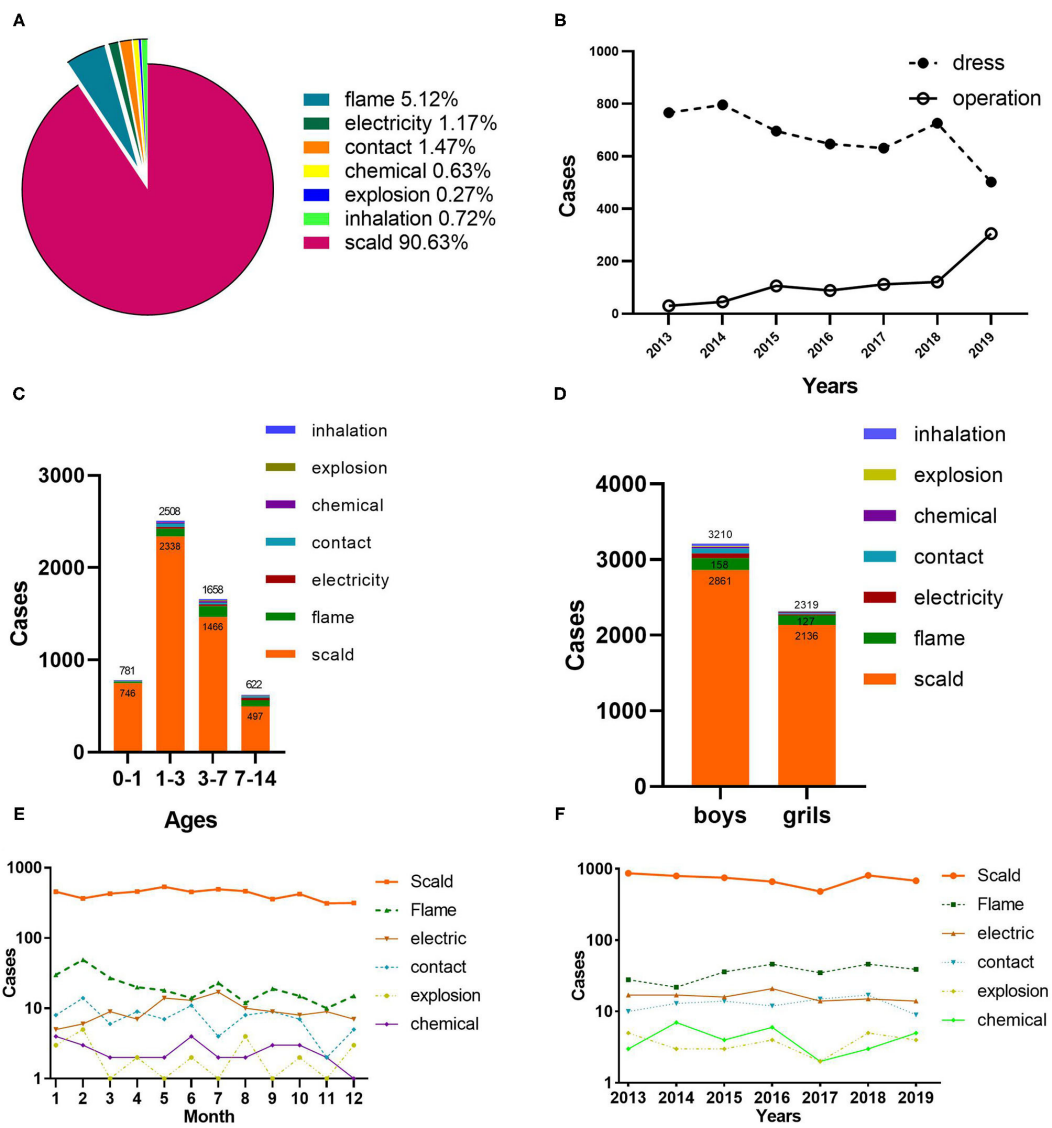
Etiology analysis and therapeutic schedule. **(A)** The proportion of different causes of burns. **(B)** Therapeutic schedule by years. **(C)** Etiology distribution by age. **(D)** Gender distribution of etiology. **(E)** Month distribution of etiology. **(F)** Distribution of etiology by years.

Scald accounted for most pediatric burns (90.63%), followed by flame (5.12%). In addition, electricity injury, thermal contact injury, chemical burns, explosion injury, and inhalation injury occupied 1.17, 1.47, 0.63, 0.27, and 0.72%, respectively (Figure 2A). 65 patients with electrical burns consist of 42 current burns and 23 arc burns. Flame and scalding were the primary causes of burns in all the age groups. Contact and electric burns were less common in the 0–1 age group, whereas they were the third and fourth causes of burns in the other groups, respectively (Figure 2C). The gender ratio was also distinct by etiology. Electric burns achieved the maximum ratio of 5.3:1.0, followed by contact burns (3.8:1.0), inhalation (3.4:1.0), explosion (2.8:1.0), and chemical (1.5:1.0). Moreover, the lowest proportions were observed for scald burns (1.3:1.0) and flames (1.2:1.0) (Figure 2D). Epidemic peaks of burns caused by different etiologies were observed during multiple periods. High-incidence periods for flames were found to be January and February, while scald burns were seen to be May, July, and August. Furthermore, July was distinguished as the peak period for electrical burns. It is noteworthy that scald burns accounted for the maximum proportion of all the months (Figure 2E). From 2013 to 2019, scald burns showed a figure of a wave. However, the trends of other etiologies fluctuated insignificantly (Figure 2F).

### Wound Treatment Methods and Surgeries

Figure 2B indicates that wound dressing was the primary treatment for burn wounds, whereas only 30 patients (3.8%) underwent surgery in 2013. In contrast, the surgical treatment significantly increased by 37.8% in 2019. Debridement and skin grafting were highly correlated with cause, age, full-thickness burns, and TBSA among children with burns, with 23.2% of all the patients undergoing surgery (Table 2). The majority of operations involved electric burns, whereas scalds were the least common (*p* < 0.01). The percentage and frequency of operations did not differ statistically significantly between boys and girls (*p* = 0.376). With the increase in TBSA, the frequency of operations and the proportion of patients undergoing operations generally increased (*P* < 0.001). The numbers of surgeries were highest in children aged 0–1 years, while surgeries percentage in children aged 7–14 years was the largest (*P* < 0.001). In the recent years, the frequency and proportion of surgeries in children with full-thickness burns have increased dramatically (*p* < 0.001).

**Table 2:** Distribution of factors related to hospitalization.

	**LOS (days) median (IQR)**	**LOS/TBSA (days)** **median (IQR)**	**Surgery (%)**	**Surgery no.** **media*n* (IQR)**	**Cured *n* (%)**	**Death** ***n*** **(%)**	**Cost (USD) media*n* (IQR)**	**Cost/TBSA (USD)** **median (IQR)**
**Etiology**								
Scald	14 (8–25)	2.1 (1.1–4.3)	484 (9.7)	0 (0–1)	4,180 (83.8)	4	1,512 (855–2,630)	242 (117–461)
Flame	18 (9.75–33)	2.4 (1–7)	55 (21.9)	0 (0–1)	206 (82.1)	3	1,451 (723–2,649)	231 (87–485)
Contact	14 (7.5–25)	3.8 (1.2–12.3)	13 (11.5)	2 (1–2)	92 (81.4)	0	1,414 (643–2,760)	446 (117–1,383)
Electric	24 (13–34)	13.5 (1.3–45.5)	38 (36.9)	1 (1–2)	86 (83.5)	0	1,831 (963–4,210)	874 (164–4,726)
Chemical	14 (7–20)	4.7 (1.3–9.5)	6 (15)	0 (0–1)	31 (77.5)	0	1,374 (872–2,380)	349 (215–1,681)
Explosion	15 (6–28.5)	1.5 (0.7–2.4)	4 (25)	0 (0–1)	8 (50)	0	1,167 (485–2,405)	201 (99–227)
*P*-value	<0.001	<0.001	<0.001	<0.001	0.103	0.012	0.131	<0.001
**Gender**								
Girls	14 (7–20)	2 (1–4)	603 (22)	0 (0–0)	2,268 (82.1)	5	1,548 (851–2,755)	238 (173–483)
Boys	15 (8–25)	2 (1–5)	678 (24.7)	0 (0–0)	2,262 (82.4)	2	1,483 (851–2,545)	254 (124–482)
*P*-value	0.535	<0.001	0.376	0.318	0.471	0.209	0.268	0.025
**Age (years)**								
0–1	15 (8–26)	2 (1–4)	141 (18)	0 (0–0)	632 (80.8)	0	1,545 (781–2,825)	240 (111–428)
1–3	15 (9–26)	2 (1–5)	701 (28.5)	0 (0–0)	2,016 (75)	5	1,412, (806–2,411)	230 (101–491)
3–7	8 (14–24)	2 (1–5)	276 (16.6)	0 (0–0)	1,378 (82.7)	1	1,436 (793–2,505)	227 (97–494)
7–14	13 (8–26)	2 (1–5)	231 (37.7)	0 (0–0)	515 (84.2)	1	222 (763–2,493)	120 (94–496)
*P*-value	0.041	0.011	<0.001	<0.001	0.250	0.100	<0.001	0.002
**TBSA**								
0–10	13 (8–24)	3 (1–6)	939 (22.4)	0 (0–0)	3,448 (82.2)	0	1,419 (818–2,427)	296 (157–580)
11–20	17 (10–28)	1 (1–2)	216 (21.4)	0 (0–0)	830 (82.3)	0	1,870 (1,112–3,614)	126 (72–255)
21–30	25 (14–42)	1 (1–2)	53 (27.6)	0 (0–0)	156 (81.3)	3	1,801 (954–6,395)	76 (39–223)
31–50	32 (20–55)	1 (1–1)	30 (41.1)	0 (0–0)	62 (84.9)	2	297 (112–640)	49 (17–113)
51–100	30 (12–69)	0 (0–1)	43 (98.6)	0 (0–2)	35 (77.8)	2	295 (151–568)	24 (11–55)
*P*-value	<0.001	<0.001	<0.001	<0.001	0.882	<0.001	<0.001	<0.001
**Full thickness burns**								
With	28 (16–43)	5.5 (2–13)	198 (39.3)	0 (0–1)	402 (79.3)	5	1,341 (733–2,379)	197 (85–469)
Without	14 (8–24)	2 (1–4)	390 (7.1)	0 (0–0)	4,126 (82.5)	2	1,826 (1,081–3,059)	299 (187–494)
*P*-value	<0.001	<0.001	<0.001	<0.001	0.005	0.416	<0.001	<0.001
**Surgery no**.								
0	13 (8–22)	2 (1–4)	0 (0)	0 (0)	3,967 (81.2)	6	1,348 (793–2,193)	216 (104–397)
1	29 (22–38)	5.7 (3.4–10.3)	297 (100)	1 (1)	268 (90.2)	1	3,629 (2,816–4,794)	722 (468–1,091)
2	38 (25–50)	5 (2.8–8.7)	173 (100)	2 (2)	152 (87.9)	0	6,292 (3,866–8,626)	740 (495–1,315)
≥3	43 (28–57)	3.3 (1.7–8.3)	157 (100)	3 (3)	141 (89.8)	0	10,569 (8,515–14,148)	950 (593–2,271)
*P*-value	<0.001	<0.001	-	-	<0.001	0.004	<0.001	<0.001

*LOS, LOS/TBSA, Surgery no., Cost, Cost/TBSA are all expressed in median (IQR: Interquartile range)*.

*Surgery, cured, and Death are all expressed in N (%)*.

*p < 0.05 were considered statistically significant*.

### Outcomes

According to this article, the number of operations and full-thickness burns could significantly affect the cure rate. However, no significant difference was found among etiologies, gender, age, and TBSAs (Table 2). The cure rate was the maximum in the 7–14-year age group, yet the minimum in the 1–3-year age group (*P* = 0.250). The cure rate showed a roughly increasing trend as the TBSA increased, but it had the minimum for the TBSA higher than 51% (*p* < 0.001). The patients without full-thickness burns achieved higher cure rates than those with full-thickness burns (*P* = 0.005). In addition, the cure rate of children undergoing surgeries had increased distinctly (*P* < 0.001). As indicated by the results of this article, the mortality was extremely low (0.1%) since only 7 of the 5,569 patients died. No significant difference was reported in the distribution of mortality for ages, genders, and etiology, whereas the difference between TBSA (P < 0.001) and frequency of operations (*P* = 0.004) was statistically significant.

### Burn Sites

Table 3 lists the distribution of the etiology and burn sites. The most common anatomical part of burns was the limb, accounting for 38.3% of hospital patients. The trunk acted as the second burn site (22.9%), followed by the head, the face, and the neck (17.4%). Diverse etiologies of burns could significantly damage different anatomical parts (*P* < 0.001). Besides the flame that principally damaged the trunk, other burns chiefly damaged the limbs. Scolding and electricity were more likely to cause hand burns. Moreover, flames and scalding also resulted in injuries to the head, face, neck, hips, feet, and perineum.

**Table 3:** Burn site distribution by etiology.

**Etiology**	**Scald**	**Flame**	**Contact**	**Chemical**	**Electricity**	**Explosion**	**Total**	***P*** **value**
Limbs	3,287	72	24	6	11	3	3,403	<0.001
Trunk	1,860	119	31	6	9	6	2,031	
Head/Face/Neck	1,458	63	4	8	5	5	1,543	
Hip/Perineum	686	14	6	3	3	1	713	
Hands	499	82	33	14	43	7	678	
Feets	453	38	7	2	6	2	508	

### Burn Severity

**Figure 3:**
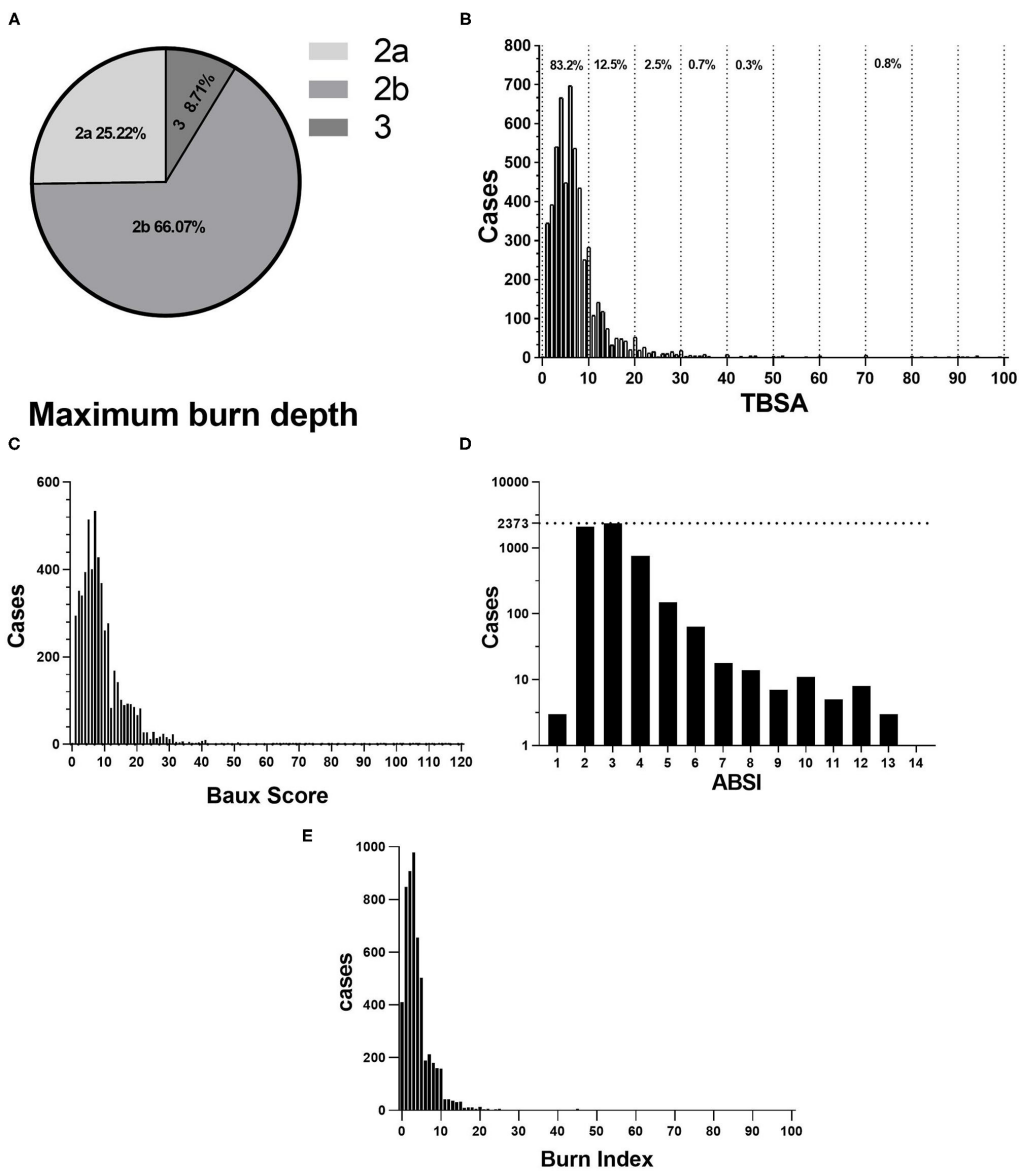
**(A)** Proportion of Maximum burn dept. 2a for superficial partial-thickness burns, 2b for deep partial-thickness burns, 3 for full-thickness burns. **(B)** Distribution of TBSA. **(C)** Distribution of Baux score. **(D)** Abbreviated Burns Severity Index (ABSI). **(E)** BI.

Most patients found deep partial-thickness and full-thickness for the maximum burn depth (Figure 3A). According to Figure 3B, the median TBSA was 6% (IQR: 4–10%). Patients with TBSA 0–10% and 11–20% accounted for 83.2 and 12.5%, respectively. Manifestations of TBSA vary markedly in etiology and age (Table 3). The Baux score ranged from 1 to 117, with a median of 7 (IQR: 4–11). According to Figure 3C, patients with a Baux score <30 were the majority (5,353/5,569, 96.12%), indicating that the mortality was not larger than 1%. The ABSI ranged from 1 to 13 and its median was 6 (IQR: 4–8). Patients with ABSI of 2–3 and 4–5 were 80.19 (4,466/5,569) and 16.30% (908/5,569) (Figure 3D), so the probability of surviving was estimated to be 99 and 98%, respectively. The average BI was 4.16 ± 5.19 (median: 3), ranging from 0 to 92. The patients with a BI of <10 accounted for 93% and 43 (5,203/5,569) (Figure 3E). The etiology was significantly correlated with the burn severity scores (*p* < 0.001; Table 4). Flame burns were significantly higher than other burns for ABSI, Baux score, and BI (*p* < 0.001; Table 4). Notably, girls had higher TBSA than boys (*p* < 0.001; Table 4). Baux scores were not statistically different with genders. The explosion and flame burns achieved the highest ABSI and Baux scores, whereas the lowest Baux score and BI were electrical burns and chemical burns, respectively, and no statistical difference was found. Obviously, the ABSI score was the highest in the 7–14-year age group (*p* < 0.001; Table 4). Moreover, among all the groups, the 3–7-year age group had the maximum BI (*p* < 0.001; Table 4). Furthermore, the ABSI and BI did not vary significantly from 2013 to 2019, whereas the TBSA and Baux scores were higher than those of other years (*p* < 0.001; Table 4).

**Table 4:** Burn severity median (IQR) display.

	**TBSA**	**ABSI**	**Baux score median**	**Burn index**
	**median (IQR)**	**median (IQR)**	**median (IQR)**	**median (IQR)**
**Etiology**				
Scald	6 (4–10)	5.5 (4.1–7.3)	7 (5–11)	3 (1–5)
Flame	6 (4–13)	7.9 (6–11.98)	7.5 (4–17)	3 (1–7)
Contact	4 (1–9)	6.1 (5–8.35)	4 (2–10)	2 (1–5)
Electric	2 (1–8)	7.1 (6–12)	3 (2–9)	1.5 (1–4)
Chemical	4 (1–7)	6 (4.45–8.70)	4 (2–8)	1 (1–4)
Explosion	8 (4–14.5)	8 (6.4–13.5)	9 (5–15.5)	3 (1–12.5)
*P* value	<0.001	<0.001	<0.7g001	<0.001
**Gender**				
Girls	7 (4–12)	6 (5–8.7)	8 (5–12)	3 (2–5.5)
Boys	6 (4–10)	6 (3.9–7)	7 (4–11)	3 (1–5)
*P* value	0.001	<0.001	0.013	<0.001
**Age (years)**				
0–1	7 (4–10.25)	4 (3–4)	8 (5–12)	3 (2–5)
1–3	6 (4–10)	4.9 (4–5.5)	7 (5–11)	3 (1–5)
3–7	7.3 (7–8.9)	4 (3–4)	8 (5–12)	14 (8–24)
7–14	6 (4–10)	14 (12–15)	7 (3–13)	3 (1–5)
*P* value	<0.001	<0.001	<0.001	<0.001
**Years**				
2013	9 (5–15)	6 (5–7)	10 (6–16)	5 (3–8)
2014	8 (4–14)	6 (5–11)	8 (5–14)	4 (2–7)
2015	7 (3–10)	7 (6–9)	8 (4–11)	4 (2–5)
2016	6 (4–9)	5 (4–7)	7 (4–10)	2 (0–3)
2017	6 (4–9)	4.8 (4–6.7)	7 (4–11)	2 (1–4)
2018	6 (4–8)	4.7 (4–6.3)	7 (4–9)	2 (1–4)
2019	6 (4–8)	4.9 (4–6.65)	7 (4–9)	3 (1–4)
*P* value	<0.001	<0.001	<0.001	<0.001

*IQR, Interquartile range*.

*p < 0.05 were considered statistically significant*.

### Risk Factors Correlated With LOS

The IQR for LOS ranged from 8 to 25 days, with a median of 15 days. Table 5 lists the distribution of LOS and LOS/TBSA. Table 6 presents the multiple linear regression of LOS-related factors. Among all of the mentioned elements, more operations could prolong LOS to the greatest extent (standardization factor = 0.273, *P* < 0.001) and the next one was larger TBSA (standardization factor = 0.152, *P* < 0.001). In accordance with the LOS/TBSA analysis results, electric burns were most prominent in the longest LOS, followed by flame burns (Table 4, *p* < 0.001). Nevertheless, the distribution of LOS and LOS/TBSA was not significantly different between genders or ages.

**Table 5:** Multiple linear regression analysis on related factors of length of stay (LOS).

	**Unstandardized** **beta coefficients**	**Standardized** **beta coefficients**	**t**	***P*** **value**	**95% CI**	
					**Lower**	**Upper**
Better outcomes	0.348	0.152	11.966	<0.001	0.291	0.405
Larger TBSA	8.444	0.044	3.455	0.001	3.653	13.234
With inhalation injury	8.850	0.125	9.396	<0.001	7.003	10.696
Full-thickness burns	7.970	0.273	20.794	<0.001	7.219	8.722
More surgeries	−6.944	−0.130	−10.513	<0.001	−8.239	−5.649

*p < 0.05 were considered statistically significant CI confidence interval*.

**Table 6:** Multiple linear regression analysis of related factors of cost.

	**Unstandardized** **beta coefficients**	**Standardized** **beta coefficients**	**t**	***P*** **value**	**95% CI**	
					**Lower**	**Upper**
Larger TBSA	200.371	0.076	5.896	<0.001	0.013	0.027
With inhalation injury	−12,798.316	−0.058	−4.550	<0.001	−1.831	−7.30
Full-thickness burns	−1,072.336	−0.013	−0.983	0.326	−3,211.592	1,066.920
More surgeries	5,933.437	0.177	12.971	<0.001	0.504	0.684
Better outcomes	−1,608.680	−0.026	−2.095	0.036	−0.309	−0.008
Longer length of stay	344.552	0.299	22.131	<0.001	0.031	0.038

*p < 0.05 were considered statistically significant*.

### Factors Correlated With Treatment Costs

The inpatient cost IQR was 845–2639 USD (median: 1,506 USD). Table 2 illustrates the cost and the cost/TBSA distribution. Table 6 lists the results of multiple linear regression in the cost analysis. Medical expenses increased with the length of hospital stay (standardization factor = 0.99, *P* < 0.001). With the increase in the number of operations (standardization factor = 0.177, *P* < 0.001), the statistical results revealed the statistically significant difference (*P* < 0.001), the cost of medical gradually increased, followed by larger TBSAs (standardization factor = 0.076, *P* < 0.001). Among the different etiology of burns, electric burns cost and cost/TBSA were more than those of other burns. Furthermore, the cost of patients with full-thickness burns was significantly higher than that of patients without it (Table 2; *p* < 0.001). Besides, the cost among different ages was significantly different. The 3–7-year group was the highest, whereas no significant difference was reported between genders.

### LA50 and the Details of 7 Deaths

LA50 results indicate an upward trend with increasing age (Table 7). Table 8 shows the details of the seven deceased patients. Three cases involved flame burns, while four were scalding burns. The shortest admission time following a burn injury was 2 h, while the longest was 6 days. TBSAs were >20% in all the patients. Four patients had combined inhalation injuries and MODS was the most common cause of death.

**Table 7:** Lethal area 50 (LA50) with 95% CIs for each age group.

**Age group**	**Lethal area 50**	**95% CI**
0–3	76.32	72.63–81.47
3–7	78.51	74.35–82.73
7–14	87.37	83.45–96.75
Overall	78.63	75.12–83.45

**Table 8:** The detail of 7 deaths.

**Patient ID**	**Age**	**Gender**	**Etiology**	**Post-injury admission time**	**%TBSA**	**Full-thickness**	**Surgery no**.	**Inhalation injury**	**Death causes**
Case 1	4	Girl	Flame	2 h	22	4	1	With	ARDS MODS
Case 2	1	Boy	Scald	3 days	45	5	0	With	Sepsis MODS
Case 3	1	Boy	Scald	5 h	91	0	0	Without	Shock MODS
Case 4	1	Boy	Flame	6 days	21	4	0	With	Sepsis MODS
Case 5	1	Boy	Scald	2 days	35	4	0	Without	Sepsis MODS
Case 6	8	Girl	Flame	8 h	63	34	0	With	ARDS MODS
Case 7	2	Boy	Scald	5 days	25	0	0	Without	Sepsis MODS

*%TBSA: total body surface area burned*.

## Discussion

The cognitive capacity of children for understanding risk factors is inadequate. The risks they face daily may result in burns. Severe burns can permanently harm a child's body, causing crippler or death (19). Consequently, an epidemiological survey of pediatric burns should be conducted. The critical link is how to evaluate the efficacy of existing burn precautionary measures and implement effective personalized prevention methods (20). However, central China's comprehensive investigation and analysis have not been carried out. This study was undertaken to investigate the epidemiology of pediatric burns in central China from 2013 to 2019 and to examine relevant aspects of clinical treatment, with the goal of developing individualized preventative and therapeutic strategies. Moreover, our research results indicated that pediatric burns patients accounted for 43.9% of the total burn patients, higher than the proportions reported previously by other centers (8, 10, 21–23). The mentioned change could be attributed to the fact that the number of burns remained high over the past 7 years as well as a lack of awareness about the prevention of scald burns. Prevention strategies are formulated by first identifying the risk factors that cause burns, then summarizing the appropriate guidelines, and finally avoiding the risks (24). In this study, there was a high prevalence of pneumonia, with the underlying cause being the loss of the natural skin barrier and the immune system being dysregulated due to burns, resulting in high infection rates (25). Uniformly, volatile fluctuations (e.g., pneumonia morbidity, shock rates, inhalation injury rates, and cure rates) were typically observable and, to some extent, unpredictable so that prevention strategies could have been modified according to the existing conditions.

The etiology of injuries consists of scald, flame, contact, electricity, chemical, etc. In this study, 90.63% of burns were caused by scalding, significantly higher than in previous studies (24, 26, 27). High-temperature liquids (e.g., milk and water) could easily cause burns. In addition, according to the analysis of the data of this article, the second most common cause of burn was flame, comprising 5.12% of all the cases, whereas it was still lower than other existing reports (28). Coal mine gas explosions, natural gas, gasoline, and other flammable substances were the most common causes of flame burns. Other than the 0–1 age group, contact burns were the third most common type of burn. Despite their rarity, electrical burns (1.17%), chemical burns (0.63%), and explosion burns (0.27%) were more serious. Hot metal, thermal friction, and stoves were the primary causes of contact burns. Based on the mentioned findings, individualized preventive strategies for different types of burns, especially scald burn prevention, should be implemented. Therefore, the following practical strategies could be used, i.e., when bathing children, cool the water down to <40°C (29). The water heater temperature should be set below 50°C (30) because children can be severely scalded in 2 ss if the water temperature is 65–70°C (31). Remove any potentially dangerous items that could cause burns or add protective measures and do not place a thermos bottle on the table. The heater and stove should not be accessible to children. All the sockets accessible to children should be covered with unique plastic covers. Moreover, following a burn, it is essential to take corresponding preventative measures (32, 33). Based on this study, scald injuries occurred more frequently in the winter and summer and flame injuries more often in January, February, and December. Summer and winter vacations are invariably the peak period for people to travel or visit relatives or friends, which increase the risk of electrical burns. Following the analysis, fire agencies and community organizations should enhance prevention publicity and education based on the types of burns most prevalent in specific months (34–36). However, peak times are not absolute and all the burns can occur at any time. As a result, it is critical to prevent burns at all the times (37). Accordingly, further evaluations are required concerning the environment around the home and burn prevention measures to ensure that they can reduce the incidence of pediatric burns (38).

Furthermore, pediatric burns were most common in patients aged 1–3 years. The prevalence of burns in this study was inversely related to age. Nevertheless, BI and Baux scores were positively correlated with age. Additionally, the rate of inhalation burns, LOS, LOS/TBSA, cost, cost/TBSA, curative effect, and cure rate changed with age. Physiologically, pediatric burns were broken down into the four groups: infants (≤ 1 year), young children (>1 and ≤ 3 years), preschoolers (>3 and ≤ 7 years), and school-age children (>7 and ≤ 14 years). As most parents are busy with work in this region, grandparents are the principal caregivers for kids. Despite this, the elderly disregard safety education and a lack of defensive skills, which is one of the leading causes of burn injuries. Generally speaking, burns sustained by school-aged children are significantly reduced due to an increasing understanding of burn prevention (39). Preschoolers are usually more susceptible to burns due to their great curiosity about things around them, uncoordinated movements, and lack of security awareness. Consequently, the annual incidence rate of preschool children is significantly higher than that of other groups. Based on the investigation and analysis, publicity and education on burn prevention should be performed individually according to age (40). Preschoolers should be provided with extensive safety training regarding burn prevention, and guardians' awareness of prevention must be consistently reinforced to deepen and consolidate prevention knowledge (41). By sharing educational materials with guardians, preschool teachers, community workers, and rural committee members can alert them to prevent burns among school-age children. During their visits to kindergartens, elementary schools, and middle schools, professional firefighters should emphasize the importance of burn prevention education for children and teachers. As a result, the government has enacted legislation and regulations aimed at reducing burn injuries (e.g., regular inspections of natural gas leaks in houses, as well as mandatory training for burn prevention in the community).

Surgery is a reliable means of removing necrotic tissue from the wound, thus keeping the patient's prognosis positive. As observed in the research results of this article, surgery has increased in recent years. Comparatively, active surgical treatment increased the cure rate by nearly 9%. It is noteworthy that pediatric burns mainly resulted from scalds, and the local vascular congestion of burn areas was gradually damaged. This resulted in most of the wounds being deep and the second-degree burns. Moreover, most burn patients' parents choose conservative dressings unless the burn is a full-thickness burn. Besides, as revealed by the statistical analysis, patients with full-thickness burns underwent surgery 20.2% more frequently than those without full-thickness burns. Recent studies have shown that timely removing necrotic skin could reduce costs, shorten healing times, and achieve more beneficial outcomes (42, 43). Likewise, such burns with partial thickness were initially treated by surgery in our center rather than conservatively. After the necrotic epidermis and part of the dermis were removed, a biological dressing was applied to the wound. By adopting this treatment strategy, we would be able to completely debride the wound, reduce the inflammation of the wound, shorten the wound healing time, and avoid the pain associated with conservative dressing. As a consequence, this study was inconsistent with prior research (44), whereas it achieved favorable clinical results. Consequently, to treat burn wounds more accurately, a healthy living environment should be created through the formulation of public policies (45), and clinical evidence-based programs should be encouraged.

The outcomes of clinical treatment are affected by many factors (46). Findings in this study indicated that the cure rate was not related to age and gender, but performed strongly positive correlations with etiology, TBSA, surgery, and full-thickness burns. The following points can illustrate the mentioned results. First, burns of all the types, full-thickness burns, a greater TBSA, or more surgeries generally indicated a higher burn severity and tended to fulfill the operation indications quite easily. Second, patients with severe burns were in unstable condition and even life-threatening, so they usually chose to be completely cured before being discharged, while patients with relatively mild burns tended to be discharged for outpatient treatment. This article indicated that mortality was significantly correlated with surgery and TBSA, but only slightly with other factors. According to statistics, the mortality rate was 0.1%, lower than other previous studies (25, 47). The total LA50 in this study was 78.63%, which is similar to previous studies (48). Explaining the situations below can illustrate the phenomenon above. On the one hand, it is generally acknowledged that inhalation injuries, extensive burns, severe shock, and infection are high-risk factors that significantly impact mortality. Our burn center has substantially reduced mortality for patients with severe burns by establishing a burn intensive care unit and other measures to monitor and treat them more effectively. On the other hand, patients without inhalation injury accounted for 99.2% in this study, whereas only 2.1% had a TBSA above 30%, which may account for the lower mortality rate than in other studies (8, 23, 49, 50). Additionally, our burn center has significantly improved the level of pediatric burn diagnosis and treatment.

The median cost was 1,511 USD in this study (IQR: 848–2,648 USD) (average: 2,299 ± 3,699 USD), differing from that recorded in Chongqing (9). These differences may be attributed to the regional economy and social-economic status, burn severity, treatment expectations, and treatment strategies. Furthermore, this article also shows that different TBSA, frequency of surgery, burn depth, inhalation injury, outcome, and LOS are correlated with cost, but not with age or gender, which can be explained as follows. First, the larger the burn area, the more complicated the treatment will be, and full-thickness burns require multiple surgical treatments, resulting in high treatment costs. Second, inhalation injury significantly increased the risk of pulmonary infection, contributing to sepsis. Flames or chemicals can cause inhalation injury that is more susceptible to pulmonary infection, one of the causes of sepsis. For this reason, comprehensive treatment strategies are usually more expensive and cost-consuming. Third, the parents preferred to discharge until the wound healed. In addition, the median LOS was 15 days (IQR: 8–25 days), which approximated the findings of existing studies (1, 24, 41, 51, 52). Analogous to cost, according to research results, LOS was associated with the etiology, TBSA, etc. Among the factors above, surgeries and deep burns were major factors in LOS. Based on the explanation of the cost above, the underlying cause may be related. Overall, patients with major burns or full-thickness burns are more likely to undergo repeated surgeries and secondary complications. Having more staff involved in their treatment and ancillary care increases cost and LOS.

The findings indicate that current pediatric burn treatment and prevention are beneficial, albeit at a low level, implying that more advanced preventative strategies should be implemented. There are limitations to this study, however, as explained below:

1. Even though our burn center is the largest in Central China with the highest number of burn hospitalizations, this study represents only the pediatric burn epidemiology in that region.
2. Outpatients were excluded from the analysis of patients, despite their higher number than inpatients.
3. There was an insufficient sample size (7 deaths) to investigate risk factors for mortality.

## Conclusion

An in-depth description of the pediatric burns epidemiology and clinical characteristics was presented in this article, which includes the number of burns in Central China, age, gender distribution, etiology, severity, complications, and surgery of pediatric burns from 2013 to 2019. Additionally, the LOS, LA50, and cost were analyzed. The following conclusions of this article can be drawn. In total, 0–3 years of age is the primary age of onset of pediatric burns. Scald burns are considered the main prevention target. Therefore, prevention and treatment strategies should be based on the risk factors above. First, education regarding safety should be gradually increased for guardians. Second, dangerous elements (e.g., scald, flame, and electricity) should be kept away from pediatrics. Third, the level of medical care for pediatric burns should be continuously improved.

## Data Availability Statement

The raw data supporting the conclusions of this article will be made available by the authors, without undue reservation.

## Ethics Statement

The studies involving human participants were reviewed and approved by the Ethical Review Committee of Zhengzhou First People's Hospital. Written informed consent from the participants' legal guardian/next of kin was not required to participate in this study in accordance with the national legislation and the institutional requirements.

## Author Contributions

ST is responsible for the design of the study. DH is responsible for the data collection and essay writing of the study. RL is responsible for the data analysis of the study. All authors contributed to the article and approved the submitted version.

## Funding

This study was funded by the Joint Co-construction Project (LHGJ20191002) of China's Henan Province Science and Technology Research Plan. However, the source of funding was not involved in the study design, data collection and analysis, writing, and article publication of this manuscript.

## Conflict of Interest

The authors declare that the research was conducted in the absence of any commercial or financial relationships that could be construed as a potential conflict of interest.

## Publisher's Note

All claims expressed in this article are solely those of the authors and do not necessarily represent those of their affiliated organizations, or those of the publisher, the editors and the reviewers. Any product that may be evaluated in this article, or claim that may be made by its manufacturer, is not guaranteed or endorsed by the publisher.

## References

[1] Lee CJ Mahendraraj K Houng A Marano M Petrone S Lee R. Pediatric Burns: A Single Institution Retrospective Review of Incidence, Etiology, and Outcomes in 2273 Burn Patients (1995-2013). J Burn Care Res. (2016) 37:e579–e85. 10.1097/BCR.0000000000000362 27294854

[2] McLeod JS Maringo AE Doyle PJ Vitale L Klein JD Shanti CM. Analysis of Electrocardiograms Associated with Pediatric Electrical Burns. J Burn Care Res. (2018) 39:65–72. 28570306 10.1097/BCR.0000000000000591

[3] Zheng Y Lin G Zhan R Qian W Yan T Sun L. Epidemiological analysis of 9,779 burn patients in China: An eight-year retrospective study at a major burn center in southwest China. Exp Ther Med. (2019) 17:2847–54. 10.3892/etm.2019.7240 30930977 PMC6425287

[4] Shah AR Liao LF. Pediatric Burn Care: Unique Considerations in Management. Clin Plast Surg. (2017) 44:603–10. 10.1016/j.cps.2017.02.017 28576249

[5] Dinesh A Polanco T Khan K Ramcharan A. Engdahl R. Our Inner-city Children Inflicted With Burns: A Retrospective Analysis of Pediatric Burn Admissions at Harlem Hospital, NY. J Burn Care Res. (2018) 39:995–9. 10.1093/jbcr/iry026 29771374

[6] Santos JV Oliveira A Costa-Pereira A Amarante J Freitas A. Burden of burns in Portugal, 2000-2013: A clinical and economic analysis of 26,447 hospitalisations. Burns. (2016) 42:891–900. 10.1016/j.burns.2016.01.017 27133714

[7] Armstrong M Wheeler KK Shi J Thakkar RK Fabia RB Groner JI. Epidemiology and trend of US pediatric burn hospitalizations, 2003-2016. Burns. (2021) 47:551–9. 10.1016/j.burns.2020.05.021 33781634

[8] Zhu L Zhang Y Liu L Jiang J Liu Y Shi F. Hospitalized pediatric burns in North China: a 10-year epidemiologic review. Burns. (2013) 39:1004–11. 10.1016/j.burns.2012.12.014 23357625

[9] Li H Wang S Tan J Zhou J Wu J Luo G. Epidemiology of pediatric burns in southwest China from 2011 to 2015. Burns. (2017) 43:1306–17. 10.1016/j.burns.2017.03.004 28372828

[10] Zhou B Zhou X Ouyang LZ Huang XY Zhang PH Zhang MH. An epidemiological analysis of paediatric burns in urban and rural areas in south central China. Burns. (2014) 40:150–6. 10.1016/j.burns.2013.04.020 23747041

[11] Van Lieshout EM Van Yperen DT Van Baar ME Polinder S Boersma D Cardon AY. Epidemiology of injuries, treatment (costs) and outcome in burn patients admitted to a hospital with or without dedicated burn centre (Burn-Pro): protocol for a multicentre prospective observational study. BMJ Open. (2018) 8:e023709. 10.1136/bmjopen-2018-023709 30446574 PMC6252702

[12] Nakae H Wada H. Characteristics of burn patients transported by ambulance to treatment facilities in Akita Prefecture, Japan. Burns. (2002) 28:73–9. 10.1016/S0305-4179(01)00063-8 11834335

[13] Osler T Glance LG Hosmer DW. Simplified estimates of the probability of death after burn injuries: extending and updating the baux score. J Trauma. (2010) 68:690–7. 10.1097/TA.0b013e3181c453b3 20038856

[14] Forster NA Zingg M Haile SR Kunzi W Giovanoli P Guggenheim M.30 years later–does the ABSI need revision? Burns. (2011) 37:958–63. 10.1016/j.burns.2011.03.009 21493008

[15] National experts consensus on prevention and treatment of burn shock (2020 version). Zhonghua Shao Shang Za Zhi. (2020) 36:786–92. Chinese. 10.3760/cma.j.cn501120-20200623-00323 32972062

[16] ISBI Practice Guidelines Committee; Steering Subcommittee; Advisory Subcommittee. ISBI Practice Guidelines for Burn Care. Burns. (2016) 42:953–1021. 10.1016/j.burns.2016.05.013 27542292

[17] Yoshino Y Ohtsuka M Kawaguchi M Sakai K Hashimoto A Hayashi M.Wound/Burn Guidelines Committee. The wound/burn guidelines - 6: Guidelines for the management of burns. J Dermatol. (2016) 43:989–1010. 10.1111/1346-8138.13288 26971391

[18] Sandora TJ Harper MB. Pneumonia in hospitalized children. Pediatr Clin North Am. (2005) 52:1059–81,viii. 10.1016/j.pcl.2005.03.004 16009257 PMC7118979

[19] Rosanova MT Stamboulian D Lede R. Risk factors for mortality in burn children. Braz J Infect Dis. (2014) 18:144–9. 10.1016/j.bjid.2013.08.004 24275369 PMC9427502

[20] Albayrak Y Temiz A Albayrak A Peksoz R Albayrak F Tanrikulu Y. retrospective analysis of 2713 hospitalized burn patients in a burns center in Turkey. Ulus Travma Acil Cerrahi Derg. (2018) 24:25–30. 10.5505/tjtes.2017.82342 29350364

[21] Wang Y Yu X Qian W Zhou D Yang T Wang S. Epidemiologic Investigation of Chemical Burns in Southwestern China from 2005 to 2016. J Burn Care Res. (2018) 39:1006–16. 10.1093/jbcr/iry032 29939259

[22] Fan X Ma B Zeng D Fang X Li H Xiao S. Burns in a major burns center in East China from 2005 to 2014: Incidence and outcome. Burns. (2017) 43:1586–95. 10.1016/j.burns.2017.01.033 28855061

[23] Fomukong NH Mefire AC Beyiha G Lawrence M Edgar MML Nkfusai NC. Predictors of mortality of pediatric burn injury in the Douala General Hospital, Cameroon. Pan Afr Med J. (2019) 33:189. 10.11604/pamj.2019.33.189.18498 31692788 PMC6814335

[24] Trop M Herzog SA Pfurtscheller K Hoebenreich AM Schintler MV Stockenhuber A. The past 25 years of pediatric burn treatment in Graz and important lessons been learned. An overview. Burns. (2015) 41:714–20. 10.1016/j.burns.2014.10.001 25678085

[25] Lachiewicz AM Hauck CG Weber DJ Cairns BA van Duin D. Bacterial Infections After Burn Injuries: Impact of Multidrug Resistance. Clin Infect Dis. (2017) 65:2130–6. 10.1093/cid/cix682 29194526 PMC5850038

[26] Saeman MR Hodgman EI Burris A Wolf SE Arnoldo BD Kowalske KJ. Epidemiology and outcomes of pediatric burns over 35 years at Parkland Hospital. Burns. (2016) 42:202–8. 10.1016/j.burns.2015.10.011 26613626

[27] Moehrlen T Landolt MA Meuli M Moehrlen U. Non intentional burns in children: Analyzing prevention and acute treatment in a highly developed country. Burns. (2019) 45:1908–17. 10.1016/j.burns.2019.05.018 31601428

[28] Cheng W Shen C Zhao D Zhang H Tu J Yuan Z. The epidemiology and prognosis of patients with massive burns: a multicenter study of 2483 cases. Burns. (2019) 45:705–16. 10.1016/j.burns.2018.08.008 30837206

[29] Martin NA Falder S. A review of the evidence for threshold of burn injury. Burns. (2017) 43:1624–39. 10.1016/j.burns.2017.04.003 28536038

[30] Abraham JP Plourde B Vallez L Stark J Diller KR. Estimating the time and temperature relationship for causation of deep-partial thickness skin burns. Burns. (2015) 41:1741–7. 10.1016/j.burns.2015.06.002 26188899

[31] Bentivegna K McCollum S Wu R Hunter AA. A state-wide analysis of pediatric scald burns by tap water, 2016-2018. Burns. (2020) 46:1805–12. 10.1016/j.burns.2020.06.009 32646547

[32] Asena M Akelma H Salik F Karahan ZA. The seasonal and monthly distribution of body limbs affected by burns in paediatric patients in southeast Turkey. Int Wound J. (2019) 16:1273–80. 10.1111/iwj.13178 31419055 PMC7949348

[33] Elrod J Schiestl CM Mohr C Landolt MA. Incidence, severity and pattern of burns in children and adolescents: An epidemiological study among immigrant and Swiss patients in Switzerland. Burns. (2019) 45:1231–41. 10.1016/j.burns.2019.02.009 31097353

[34] Mata Ribeiro L Vieira LG Sousa JM Guerra AS. Seasonal impact in burn profiles in a dedicated burn unit. Burns. (2019) 45:1189–98. 10.1016/j.burns.2019.03.008 30948279

[35] Johnson SA Shi J Groner JI Thakkar RK Fabia R Besner GE. Inter-facility transfer of pediatric burn patients from US Emergency Departments. Burns. (2016) 42:1413–22. 10.1016/j.burns.2016.06.024 27554628 PMC5056153

[36] Mason SA Nathens AB Byrne JP Gonzalez A Fowler R Karanicolas PJ. Trends in the epidemiology of major burn injury among hospitalized patients: A population-based analysis. J Trauma Acute Care Surg. (2017) 83:867–74. 10.1097/TA.0000000000001586 28538640 PMC5656518

[37] Duke JM Boyd JH Rea S Randall SM Wood FM. Long-term mortality among older adults with burn injury: a population-based study in Australia. Bull World Health Organ. (2015) 93:400–6. 10.2471/BLT.14.149146 26240461 PMC4450710

[38] Lehna C Furmanek S Fahey E Hanchette C. Geographic modeling for children at risk for home fires and burns. Burns. (2018) 44:201–9. 10.1016/j.burns.2017.07.007 28811054

[39] Kai-Yang L Zhao-Fan X Luo-Man Z Yi-Tao J Tao T Wei W. Epidemiology of pediatric burns requiring hospitalization in China: a literature review of retrospective studies. Pediatrics. (2008) 122:132–42. 10.1542/peds.2007-1567 18595996

[40] Bousema S Stas HG van de Merwe MH Oen IM Baartmans MG van Baar ME. Epidemiology and screening of intentional burns in children in a Dutch burn centre. Burns. (2016) 42:1287–94. 10.1016/j.burns.2016.01.009 27211360

[41] Ryder C Mackean T Hunter K Towers K Rogers K Holland AJA. Factors contributing to longer length of stay in Aboriginal and Torres Strait Islander children hospitalised for burn injury. Inj Epidemiol. (2020) 7:52. 10.1186/s40621-020-00278-7 33012291 PMC7534159

[42] Vivo C Galeiras R del Caz MD. Initial evaluation and management of the critical burn patient. Med Intensiva. (2016) 40:49–59. 10.1016/j.medin.2015.11.010 26724246

[43] Church D Elsayed S Reid O Winston B Lindsay R. Burn wound infections. Clin Microbiol Rev. (2006) 19:403–34. 10.1128/CMR.19.2.403-434.2006 16614255 PMC1471990

[44] Griffin BR Frear CC Babl F Oakley E Kimble RM. Cool Running Water First Aid Decreases Skin Grafting Requirements in Pediatric Burns: a Cohort Study of Two Thousand Four Hundred Ninety-five Children. Ann Emerg Med. (2020) 75:75–85. 10.1016/j.annemergmed.2019.06.028 31474480

[45] Molster CM Bowman FL Bilkey GA Cho AS Burns BL Nowak KJ. The Evolution of Public Health Genomics: Exploring Its Past, Present, and Future. Front Public Health. (2018) 6:247. 10.3389/fpubh.2018.00247 30234091 PMC6131666

[46] Cornet PA Niemeijer AS Figaroa GD van Daalen MA Broersma TW van Baar ME. Clinical outcome of patients with self-inflicted burns. Burns. (2017) 43:789–95. 10.1016/j.burns.2016.11.005 28065425

[47] Tripathee S Basnet SJ. Epidemiology of burn injuries in Nepal: a systemic review. Burns Trauma. (2017) 5:10. 10.1186/s41038-017-0075-y 28413803 PMC5389177

[48] Ahmadabadi A Khadem-Rezaiyan M Tavousi SH. Reply to comments on “Lethal area 50 percent (LA50) or standardized mortality ratio (SMR): Which one is more conclusive?”. Burns. (2020) 46:1482–3. 10.1016/j.burns.2020.04.019 32387099

[49] Chong HP Quinn L Cooksey R Molony D Jeeves A Lodge M. Mortality in paediatric burns at the Women's and Children's Hospital (WCH), Adelaide, South Australia: 1960-2017. Burns. (2020) 46:207–12. 10.1016/j.burns.2019.06.004 31787476

[50] Sadeghi-Bazargani H Mohammadi R. Unintentional domestic burns in Iran: Analysis of 125,000 cases from a national register. Burns. (2013) 39:1304–10. 10.1016/j.burns.2013.02.013 23684319

[51] Duan WQ Xu XW Cen Y Xiao HT Liu XX Liu Y. Epidemiologic Investigation of Burn Patients in Sichuan Province, China. Med Sci Monit. (2019) 25:872–9. 10.12659/MSM.912821 30699102 PMC6364455

[52] Bailey ME Sagiraju HKR Mashreky SR Alamgir H. Epidemiology and outcomes of burn injuries at a tertiary burn care center in Bangladesh. Burns. (2019) 45:957–63. 10.1016/j.burns.2018.12.011 30612889

